# Bis(1-ferrocenylmethyl­idene-4-phenyl­thiosemicarbazidato-κ^2^ *N* ^1^,*S*)zinc(II) monohydrate

**DOI:** 10.1107/S1600536809046078

**Published:** 2009-11-07

**Authors:** M. R. Vikneswaran, Siang Guan Teoh, Chin Sing Yeap, Hoong-Kun Fun

**Affiliations:** a School of Chemical Sciences, Universiti Sains Malaysia, 11800 USM, Penang, Malaysia; b X-ray Crystallography Unit, School of Physics, Universiti Sains Malaysia, 11800 USM, Penang, Malaysia

## Abstract

In the title compound, [Fe_2_Zn(C_5_H_5_)_2_(C_13_H_11_N_3_S)_2_]·H_2_O, the Zn^II^ ion is in a distorted tetra­hedral geometry being coordinated by two thio­semicarbazone ligands *via* N and S atoms. One of the Cp rings is disordered over two positions with occupancies of 0.55 and 0.45. The dihedral angle between the substituted Cp rings is 56.1 (5)° and the two phenyl rings are orientated at a dihedral angle of 41.7 (4)°. In the crystal structure, inter­molecular O—H⋯S, N—H⋯O and C—H⋯N hydrogen bonds link the mol­ecules into chains along the *b* axis. The structure is further consolidated by O—H⋯π inter­actions.

## Related literature

For related structures, see: Vikneswaran *et al.* (2009*a* ▶,*b* ▶). For the preparation, see: Casas *et al.* (2004 ▶). For the stability of the temperature controller used for the data collection, see: Cosier & Glazer (1986 ▶).
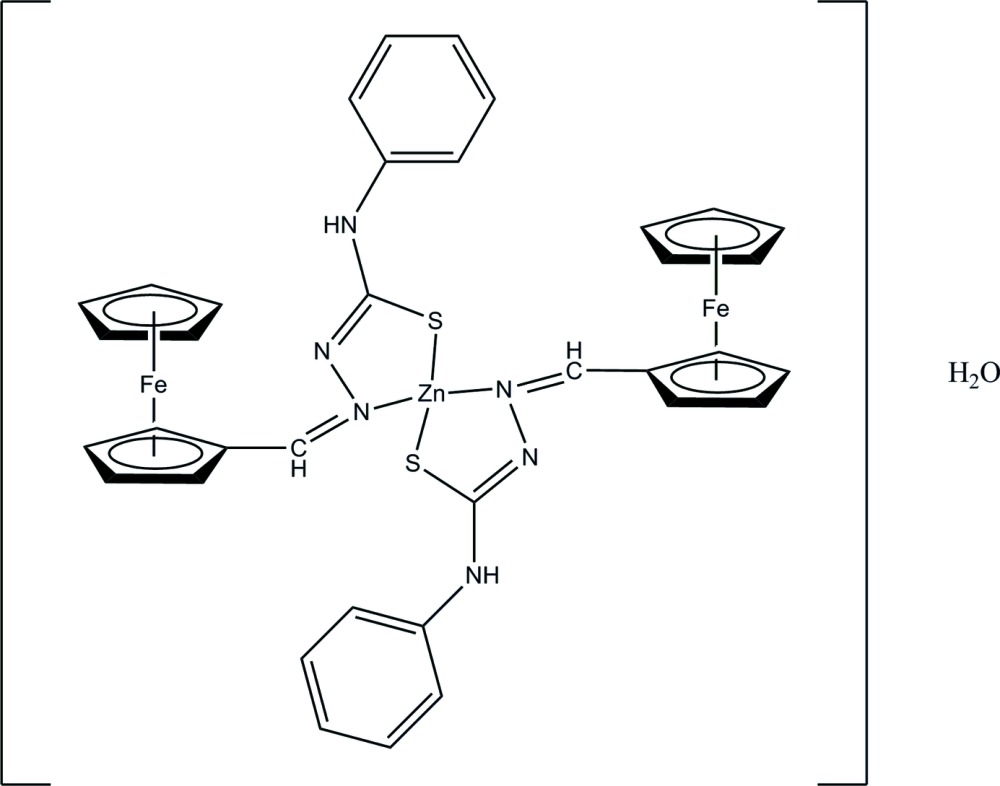


## Experimental

### 

#### Crystal data

[Fe_2_Zn(C_5_H_5_)_2_(C_13_H_11_N_3_S)_2_]·H_2_O
*M* *_r_* = 807.88
Monoclinic, 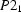

*a* = 10.4500 (5) Å
*b* = 10.4892 (5) Å
*c* = 15.9377 (7) Å
β = 104.277 (2)°
*V* = 1693.01 (14) Å^3^
*Z* = 2
Mo *K*α radiation
μ = 1.71 mm^−1^
*T* = 100 K
0.65 × 0.17 × 0.09 mm

#### Data collection

Bruker SMART APEXII CCD area-detector diffractometer
Absorption correction: multi-scan (**SADABS**; Bruker, 2005 ▶) *T*_min_ = 0.402, *T*_max_ = 0.858
17262 measured reflections
7565 independent reflections
5980 reflections with *I* > 2σ(*I*)
*R*_int_ = 0.050

#### Refinement

*R*[*F* ^2^ > 2σ(*F* ^2^)] = 0.069
*wR*(*F* ^2^) = 0.174
*S* = 1.09
7565 reflections
473 parameters
163 restraints
H-atom parameters constrained
Δρ_max_ = 2.43 e Å^−3^
Δρ_min_ = −0.71 e Å^−3^
Absolute structure: Flack (1983 ▶), 3458 Friedel pairs
Flack parameter: 0.50 (2)

### 

Data collection: *APEX2* (Bruker, 2005 ▶); cell refinement: *SAINT* (Bruker, 2005 ▶); data reduction: *SAINT*; program(s) used to solve structure: *SHELXTL* (Sheldrick, 2008 ▶); program(s) used to refine structure: *SHELXTL*; molecular graphics: *SHELXTL*; software used to prepare material for publication: *SHELXTL* and *PLATON* (Spek, 2009 ▶).

## Supplementary Material

Crystal structure: contains datablocks global, I. DOI: 10.1107/S1600536809046078/ci2959sup1.cif

Structure factors: contains datablocks I. DOI: 10.1107/S1600536809046078/ci2959Isup2.hkl

Additional supplementary materials:  crystallographic information; 3D view; checkCIF report

## Figures and Tables

**Table 1:** Hydrogen-bond geometry (Å, °)

*D*—H⋯*A*	*D*—H	H⋯*A*	*D*⋯*A*	*D*—H⋯*A*
O1*W*—H2*W*1⋯S1^i^	0.85	2.61	3.238 (10)	132
N3—H3*B*⋯O1*W* ^ii^	0.86	2.14	2.981 (10)	165
N6—H6*B*⋯O1*W*	0.86	2.12	2.927 (11)	155
C18—H18*A*⋯N2^iii^	0.98	2.60	3.455 (10)	146
O1*W*—H1*W*1⋯*Cg*1^iv^	0.85	2.63	3.164 (9)	123

Symmetry codes: (i) 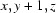; (ii) 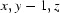; (iii) 
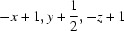
; (iv) 
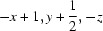
. *Cg*1 is the centroid of the C1–C5 ring.

**Fig. 1.**
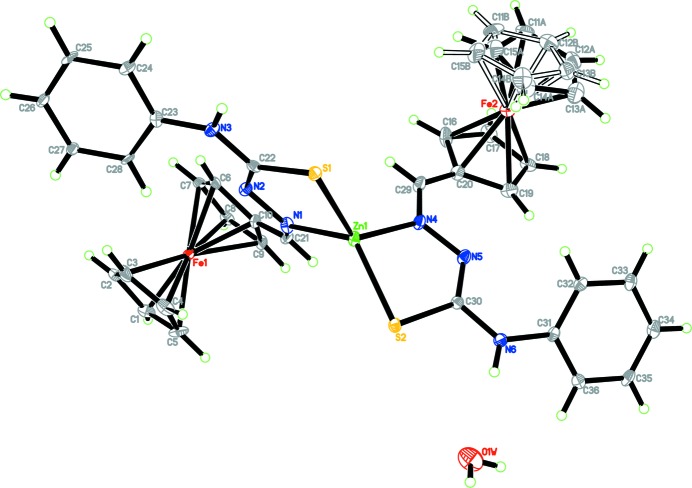
The molecular structure of the title compound, showing 50% probability displacement ellipsoids and the atom-numbering scheme. All disorder components are shown.

**Fig. 2.**
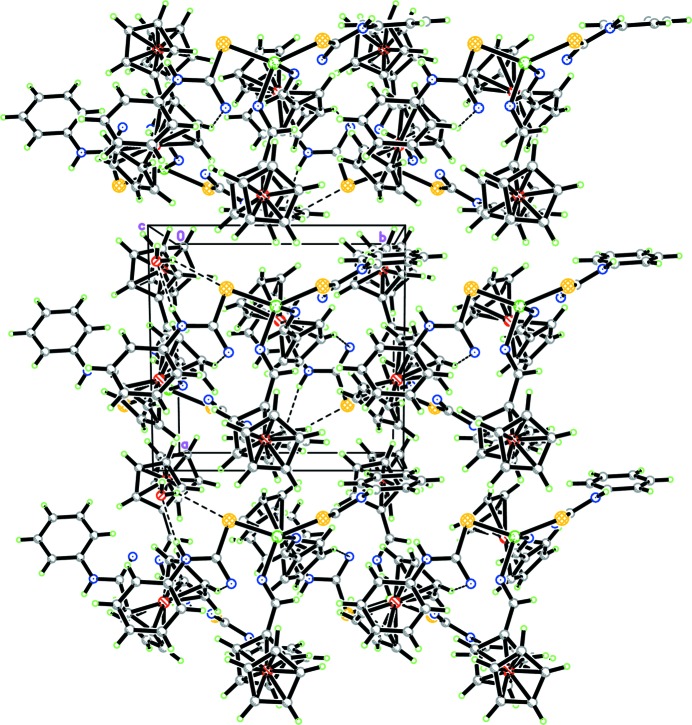
The crystal packing of the title compound, viewed along the c axis, showing chains along b axis. Intermolecular hydrogen bonds are shown as dashed lines. Only the major disorder components are shown.

## supplementary crystallographic information

### Comment

As a continuation of our research related to ferrocenyl thiosemicarbazones and its metal complexes, herein we report the synthesis and crystal structure of a Zn^II^ complex formed with formylferrocene 4-phenyllthiosemicarbazone.

The Zn^II^ ion is in a distorted tetrahedral geometry being coordinated by two thiosemicarbarzone ligands *via* the N and S atoms (Fig. 1). The bond lengths and angles are comparable to those observed in closely related structures (Vikneswaran *et al.*, 2009*a*,b). One of the Cp rings is disordered over two positions with site occupancies of 0.55 and 0.45. The Cp rings of each ferrocene residue are nearly parallel, with dihedral angles of Cp1/Cp2 [C1–C5/C6–C10] = 2.6 (5)°, Cp3/Cp5 [C11A–C15A/C16–C20] = 4.5 (14)° and Cp4/Cp5 [C11B–C15B/C16–C20] = 7.4 (16)°. The dihedral angle between the substituted Cp rings [Cp2 and Cp5] is 56.1 (5)° and the two phenyl rings [C23–C28 and C31–C36] are orientated at 41.7 (4)°.

In the crystal structure, intermolecular O—H···S, N—H···O and C—H···N hydrogen bonds (Table 1) link the molecules into chains along the *b* axis (Fig. 2). The crystal structure is further consolidated by O—H···π interactions involving the C1–C5 ring.

### Experimental

Formylferrocene 4-phenylthiosemicarbazone was prepared as described by Casas *et al.* (2004). Zn(CH_3_COO)_2_.2H_2_O (0.21 g, 1 mmol) dissolved in methanol (60 ml) was added dropwise at room temperature to a mixture of formylferrocene 4-phenylthiosemicarbazone (0.36 g, 1 mmol) and KOH (0.12 g, 2 mmol) in absolute methanol (15 ml). Amorphous orange solid separated out immediately. The suspension was stirred under reflux for 4 h and filtered. After several days, brown crystals were obtained from the filtrate.

### Refinement

One of the Cp rings is disordered over two positions with site occupancies of 0.55 and 0.45. The same U^ij^ parameters is used for the atom pair C14A/C14B, and all disordered atoms were subjected to rigid bond and similarity restraints. All H-atoms were placed in calculated positions, with C–H = 0.93–0.98 Å, N–H = 0.86 and O–H = 0.85 and refined using a riding model, with *U*_iso_(H) = 1.2 *U*_eq_(C,N) and 1.5*U*_eq_(O). The highest residual density peak is located 0.87 Å from atom Zn1 and the deepest hole is located 1.30 Å from atom Zn1.

### Crystal data

**Table:**

[Fe_2_Zn(C_5_H_5_)_2_(C_13_H_11_N_3_S)_2_]·H_2_O	*F*(000) = 828
*M**_r_* = 807.88	*D*_x_ = 1.585 Mg m^−^^3^
Monoclinic, *P*2_1_	Mo *K*α radiation, λ = 0.71073 Å
Hall symbol: P 2yb	Cell parameters from 7082 reflections
*a* = 10.4500 (5) Å	θ = 2.4–30.1°
*b* = 10.4892 (5) Å	µ = 1.71 mm^−^^1^
*c* = 15.9377 (7) Å	*T* = 100 K
β = 104.277 (2)°	Needle, brown
*V* = 1693.01 (14) Å^3^	0.65 × 0.17 × 0.09 mm
*Z* = 2	

### Data collection

**Table:**

Bruker SMART APEXII CCD area-detector diffractometer	7565 independent reflections
Radiation source: fine-focus sealed tube	5980 reflections with *I* > 2σ(*I*)
graphite	*R*_int_ = 0.050
φ and ω scans	θ_max_ = 27.5°, θ_min_ = 2.1°
Absorption correction: multi-scan (*SADABS*; Bruker, 2005)	*h* = −13→13
*T*_min_ = 0.402, *T*_max_ = 0.858	*k* = −13→13
17262 measured reflections	*l* = −20→20

### Refinement

**Table:**

Refinement on *F*^2^	Secondary atom site location: difference Fourier map
Least-squares matrix: full	Hydrogen site location: inferred from neighbouring sites
*R*[*F*^2^ > 2σ(*F*^2^)] = 0.069	H-atom parameters constrained
*wR*(*F*^2^) = 0.174	*w* = 1/[σ^2^(*F*_o_^2^) + (0.0767*P*)^2^ + 6.158*P*] where *P* = (*F*_o_^2^ + 2*F*_c_^2^)/3
*S* = 1.09	(Δ/σ)_max_ < 0.001
7565 reflections	Δρ_max_ = 2.43 e Å^−^^3^
473 parameters	Δρ_min_ = −0.71 e Å^−^^3^
163 restraints	Absolute structure: Flack (1983), 3458 Friedel pairs
Primary atom site location: structure-invariant direct methods	Flack parameter: 0.50 (2)

### Special details

**Table:**

Experimental. The crystal was placed in the cold stream of an Oxford Cyrosystems Cobra open-flow nitrogen cryostat (Cosier & Glazer, 1986) operating at 100.0 (1) K.
Geometry. All e.s.d.'s (except the e.s.d. in the dihedral angle between two l.s. planes) are estimated using the full covariance matrix. The cell e.s.d.'s are taken into account individually in the estimation of e.s.d.'s in distances, angles and torsion angles; correlations between e.s.d.'s in cell parameters are only used when they are defined by crystal symmetry. An approximate (isotropic) treatment of cell e.s.d.'s is used for estimating e.s.d.'s involving l.s. planes.
Refinement. Refinement of *F*^2^ against ALL reflections. The weighted *R*-factor *wR* and goodness of fit *S* are based on *F*^2^, conventional *R*-factors *R* are based on *F*, with *F* set to zero for negative *F*^2^. The threshold expression of *F*^2^ > σ(*F*^2^) is used only for calculating *R*-factors(gt) *etc*. and is not relevant to the choice of reflections for refinement. *R*-factors based on *F*^2^ are statistically about twice as large as those based on *F*, and *R*- factors based on ALL data will be even larger.

### Fractional atomic coordinates and isotropic or equivalent isotropic displacement parameters (Å^2^)

**Table:**

	*x*	*y*	*z*	*U*_iso_*/*U*_eq_	Occ. (<1)
Zn1	0.68442 (7)	−0.01485 (8)	0.26651 (5)	0.02324 (19)	
Fe1	0.12446 (9)	−0.05464 (9)	0.06968 (6)	0.0193 (2)	
Fe2	0.63054 (10)	−0.01196 (12)	0.62280 (7)	0.0290 (2)	
S1	0.75745 (19)	−0.21576 (18)	0.25244 (13)	0.0247 (4)	
S2	0.7687 (2)	0.17743 (18)	0.24276 (12)	0.0252 (4)	
N1	0.5012 (5)	−0.0784 (6)	0.2047 (4)	0.0243 (14)	
N2	0.4886 (6)	−0.2052 (6)	0.1768 (4)	0.0237 (13)	
N3	0.5994 (6)	−0.3911 (6)	0.1667 (4)	0.0255 (13)	
H3B	0.6710	−0.4327	0.1880	0.031*	
N4	0.6628 (6)	0.0603 (6)	0.3798 (4)	0.0271 (14)	
N5	0.7259 (7)	0.1747 (6)	0.4074 (4)	0.0288 (14)	
N6	0.8466 (7)	0.3400 (6)	0.3717 (4)	0.0290 (14)	
H6B	0.8763	0.3752	0.3315	0.035*	
C1	0.0105 (9)	0.0322 (9)	−0.0384 (6)	0.0346 (19)	
H1A	−0.0719	0.0784	−0.0410	0.042*	
C2	0.0203 (11)	−0.0972 (9)	−0.0521 (5)	0.041 (2)	
H2A	−0.0525	−0.1582	−0.0687	0.049*	
C3	0.1618 (11)	−0.1233 (10)	−0.0410 (5)	0.043 (3)	
H3A	0.2006	−0.2066	−0.0476	0.052*	
C4	0.2298 (8)	−0.0139 (11)	−0.0172 (5)	0.040 (2)	
H4A	0.3260	−0.0046	−0.0043	0.048*	
C5	0.1380 (10)	0.0859 (9)	−0.0156 (7)	0.042 (2)	
H5A	0.1595	0.1756	−0.0014	0.051*	
C6	0.1992 (8)	−0.1840 (7)	0.1668 (4)	0.0254 (16)	
H6A	0.2461	−0.2631	0.1607	0.031*	
C7	0.0600 (8)	−0.1709 (8)	0.1560 (5)	0.0258 (16)	
H7A	−0.0055	−0.2393	0.1407	0.031*	
C8	0.0331 (7)	−0.0438 (8)	0.1694 (4)	0.0277 (17)	
H8A	−0.0550	−0.0082	0.1651	0.033*	
C9	0.1525 (8)	0.0270 (8)	0.1892 (5)	0.0284 (17)	
H9A	0.1612	0.1184	0.2021	0.034*	
C10	0.2585 (7)	−0.0599 (8)	0.1873 (4)	0.0228 (14)	
C11A	0.664 (3)	−0.157 (2)	0.7124 (14)	0.046 (5)	0.55
H11A	0.5996	−0.2050	0.7354	0.055*	0.55
C12A	0.739 (2)	−0.047 (2)	0.7480 (12)	0.050 (5)	0.55
H12A	0.7269	−0.0007	0.7988	0.059*	0.55
C13A	0.823 (2)	−0.009 (3)	0.7001 (18)	0.059 (5)	0.55
H13A	0.8817	0.0651	0.7116	0.070*	0.55
C14A	0.806 (5)	−0.090 (5)	0.629 (3)	0.048 (4)	0.55
H14A	0.8541	−0.0846	0.5832	0.057*	0.55
C15A	0.711 (3)	−0.180 (3)	0.6317 (16)	0.053 (5)	0.55
H15A	0.6833	−0.2510	0.5910	0.064*	0.55
C11B	0.623 (3)	−0.194 (2)	0.671 (2)	0.048 (5)	0.45
H11B	0.5449	−0.2435	0.6731	0.057*	0.45
C12B	0.696 (3)	−0.106 (3)	0.7340 (16)	0.046 (6)	0.45
H12B	0.6720	−0.0847	0.7882	0.056*	0.45
C13B	0.801 (3)	−0.058 (3)	0.7100 (17)	0.045 (5)	0.45
H13B	0.8650	0.0023	0.7433	0.055*	0.45
C14B	0.804 (6)	−0.112 (6)	0.630 (3)	0.048 (4)	0.45
H14B	0.8687	−0.0911	0.5971	0.057*	0.45
C15B	0.700 (3)	−0.197 (3)	0.603 (2)	0.048 (5)	0.45
H15B	0.6789	−0.2462	0.5490	0.058*	0.45
C16	0.4511 (8)	0.0066 (11)	0.5369 (5)	0.044 (2)	
H16A	0.3927	−0.0633	0.5105	0.052*	
C17	0.4488 (8)	0.0706 (10)	0.6144 (6)	0.040 (2)	
H17A	0.3898	0.0506	0.6518	0.048*	
C18	0.5481 (10)	0.1664 (8)	0.6309 (5)	0.035 (2)	
H18A	0.5690	0.2243	0.6807	0.042*	
C19	0.6136 (10)	0.1610 (8)	0.5606 (6)	0.038 (2)	
H19A	0.6871	0.2152	0.5545	0.045*	
C20	0.5551 (8)	0.0624 (8)	0.5032 (5)	0.0302 (17)	
C21	0.3959 (7)	−0.0207 (8)	0.2059 (4)	0.0255 (14)	
H21A	0.4089	0.0644	0.2221	0.031*	
C22	0.5997 (7)	−0.2673 (7)	0.1942 (4)	0.0213 (14)	
C23	0.5015 (7)	−0.4607 (7)	0.1095 (5)	0.0223 (16)	
C24	0.5092 (8)	−0.5925 (7)	0.1105 (6)	0.0274 (17)	
H24A	0.5747	−0.6329	0.1521	0.033*	
C25	0.4193 (8)	−0.6661 (7)	0.0495 (6)	0.0302 (19)	
H25A	0.4245	−0.7546	0.0515	0.036*	
C26	0.3228 (8)	−0.6063 (7)	−0.0138 (5)	0.0253 (17)	
H26A	0.2644	−0.6541	−0.0555	0.030*	
C27	0.3147 (8)	−0.4760 (7)	−0.0141 (5)	0.0241 (16)	
H27A	0.2491	−0.4358	−0.0558	0.029*	
C28	0.4022 (8)	−0.4026 (7)	0.0464 (5)	0.0222 (16)	
H28A	0.3946	−0.3142	0.0449	0.027*	
C29	0.5868 (8)	0.0136 (8)	0.4260 (5)	0.0324 (18)	
H29A	0.5465	−0.0634	0.4061	0.039*	
C30	0.7787 (7)	0.2288 (7)	0.3500 (5)	0.0225 (15)	
C31	0.8740 (8)	0.4042 (7)	0.4519 (5)	0.0243 (17)	
C32	0.8949 (8)	0.3425 (8)	0.5310 (5)	0.0253 (17)	
H32A	0.8934	0.2539	0.5331	0.030*	
C33	0.9182 (8)	0.4126 (8)	0.6074 (6)	0.0302 (19)	
H33A	0.9284	0.3714	0.6603	0.036*	
C34	0.9261 (9)	0.5440 (9)	0.6041 (6)	0.033 (2)	
H34A	0.9436	0.5916	0.6549	0.040*	
C35	0.9081 (9)	0.6034 (8)	0.5260 (6)	0.034 (2)	
H35A	0.9130	0.6918	0.5242	0.041*	
C36	0.8830 (8)	0.5354 (8)	0.4500 (5)	0.0267 (17)	
H36A	0.8721	0.5777	0.3975	0.032*	
O1W	0.8714 (7)	0.5034 (10)	0.2277 (5)	0.071 (2)	
H1W1	0.9316	0.5116	0.2007	0.106*	
H2W1	0.8786	0.5651	0.2632	0.106*	

### Atomic displacement parameters (Å^2^)

**Table:**

	*U*^11^	*U*^22^	*U*^33^	*U*^12^	*U*^13^	*U*^23^
Zn1	0.0222 (4)	0.0288 (4)	0.0219 (4)	−0.0055 (4)	0.0114 (3)	−0.0074 (4)
Fe1	0.0191 (5)	0.0176 (5)	0.0253 (5)	−0.0014 (4)	0.0134 (4)	−0.0020 (4)
Fe2	0.0297 (5)	0.0316 (5)	0.0304 (5)	0.0039 (5)	0.0166 (4)	−0.0003 (5)
S1	0.0212 (9)	0.0285 (10)	0.0244 (10)	−0.0052 (7)	0.0058 (7)	0.0007 (7)
S2	0.0308 (11)	0.0247 (9)	0.0213 (9)	−0.0009 (8)	0.0088 (8)	−0.0051 (7)
N1	0.011 (3)	0.041 (4)	0.024 (3)	−0.005 (3)	0.010 (2)	−0.006 (3)
N2	0.028 (3)	0.023 (3)	0.024 (3)	−0.002 (3)	0.015 (3)	−0.005 (2)
N3	0.022 (3)	0.021 (3)	0.034 (3)	0.001 (3)	0.009 (3)	−0.002 (3)
N4	0.029 (3)	0.028 (4)	0.026 (3)	−0.008 (3)	0.011 (3)	−0.009 (3)
N5	0.033 (4)	0.025 (3)	0.030 (3)	−0.003 (3)	0.011 (3)	−0.008 (3)
N6	0.042 (4)	0.026 (3)	0.021 (3)	−0.006 (3)	0.013 (3)	−0.004 (3)
C1	0.030 (4)	0.041 (5)	0.038 (5)	0.009 (4)	0.018 (4)	0.011 (4)
C2	0.064 (7)	0.041 (5)	0.018 (4)	−0.011 (4)	0.010 (4)	0.004 (3)
C3	0.065 (7)	0.041 (5)	0.024 (4)	0.024 (5)	0.011 (4)	0.000 (4)
C4	0.032 (4)	0.063 (6)	0.033 (4)	0.010 (5)	0.022 (3)	0.007 (5)
C5	0.053 (6)	0.024 (4)	0.059 (6)	−0.001 (4)	0.031 (5)	0.012 (4)
C6	0.030 (4)	0.030 (4)	0.017 (3)	−0.003 (3)	0.007 (3)	0.001 (3)
C7	0.034 (4)	0.027 (4)	0.021 (4)	−0.003 (3)	0.014 (3)	0.003 (3)
C8	0.019 (3)	0.043 (5)	0.025 (3)	0.002 (3)	0.012 (3)	0.005 (3)
C9	0.034 (4)	0.025 (4)	0.026 (4)	0.007 (3)	0.009 (3)	−0.009 (3)
C10	0.019 (3)	0.027 (3)	0.024 (3)	0.000 (3)	0.008 (3)	−0.006 (3)
C11A	0.082 (14)	0.028 (12)	0.028 (10)	0.015 (8)	0.017 (9)	0.008 (7)
C12A	0.067 (12)	0.038 (12)	0.034 (7)	0.026 (9)	−0.005 (7)	−0.005 (7)
C13A	0.043 (10)	0.044 (13)	0.077 (11)	0.013 (8)	−0.007 (8)	−0.014 (10)
C14A	0.029 (4)	0.055 (13)	0.060 (5)	0.018 (7)	0.012 (4)	−0.009 (7)
C15A	0.072 (12)	0.042 (11)	0.053 (12)	0.009 (8)	0.030 (11)	−0.013 (9)
C11B	0.050 (11)	0.021 (10)	0.073 (15)	0.009 (8)	0.016 (10)	0.009 (10)
C12B	0.054 (14)	0.045 (18)	0.043 (11)	0.019 (11)	0.019 (10)	0.003 (10)
C13B	0.045 (11)	0.047 (15)	0.040 (9)	0.001 (9)	0.002 (8)	−0.001 (10)
C14B	0.029 (4)	0.055 (13)	0.060 (5)	0.018 (7)	0.012 (4)	−0.009 (7)
C15B	0.051 (10)	0.031 (11)	0.061 (14)	0.014 (7)	0.010 (11)	−0.019 (11)
C16	0.032 (4)	0.073 (7)	0.030 (4)	−0.015 (5)	0.014 (3)	−0.016 (5)
C17	0.026 (4)	0.064 (6)	0.037 (5)	0.005 (4)	0.019 (4)	−0.003 (4)
C18	0.058 (6)	0.028 (4)	0.025 (4)	0.016 (4)	0.020 (4)	−0.001 (3)
C19	0.050 (5)	0.032 (5)	0.038 (5)	0.003 (4)	0.024 (4)	−0.001 (4)
C20	0.030 (4)	0.035 (4)	0.030 (4)	−0.002 (3)	0.015 (3)	−0.005 (3)
C21	0.032 (4)	0.020 (3)	0.028 (3)	−0.005 (3)	0.013 (3)	−0.004 (3)
C22	0.017 (3)	0.028 (4)	0.019 (3)	0.000 (3)	0.006 (3)	0.006 (3)
C23	0.013 (4)	0.036 (4)	0.023 (4)	−0.007 (3)	0.013 (3)	−0.006 (3)
C24	0.028 (4)	0.019 (4)	0.038 (5)	0.000 (3)	0.014 (4)	−0.001 (3)
C25	0.042 (5)	0.007 (3)	0.046 (5)	0.005 (3)	0.020 (4)	−0.005 (3)
C26	0.032 (4)	0.017 (3)	0.029 (4)	−0.005 (3)	0.012 (3)	−0.005 (3)
C27	0.033 (4)	0.018 (4)	0.024 (4)	−0.004 (3)	0.013 (3)	−0.004 (3)
C28	0.030 (4)	0.010 (3)	0.030 (4)	−0.007 (3)	0.014 (3)	−0.002 (3)
C29	0.036 (4)	0.032 (5)	0.032 (4)	−0.011 (3)	0.015 (3)	−0.011 (3)
C30	0.026 (4)	0.020 (3)	0.022 (4)	0.002 (3)	0.007 (3)	−0.003 (3)
C31	0.025 (4)	0.019 (3)	0.029 (4)	0.002 (3)	0.009 (3)	−0.010 (3)
C32	0.025 (4)	0.020 (4)	0.032 (4)	0.003 (3)	0.009 (3)	−0.003 (3)
C33	0.014 (4)	0.038 (5)	0.037 (5)	−0.004 (3)	0.005 (3)	−0.008 (4)
C34	0.033 (5)	0.039 (5)	0.032 (5)	−0.003 (4)	0.015 (4)	−0.017 (4)
C35	0.038 (5)	0.025 (4)	0.042 (5)	−0.010 (3)	0.013 (4)	−0.015 (4)
C36	0.019 (4)	0.037 (4)	0.026 (4)	0.000 (3)	0.009 (3)	0.000 (3)
O1W	0.067 (5)	0.091 (7)	0.062 (4)	0.013 (5)	0.031 (4)	0.006 (5)

### Geometric parameters (Å, °)

**Table:**

Zn1—N4	2.033 (6)	C9—H9A	0.98
Zn1—N1	2.038 (6)	C10—C21	1.452 (9)
Zn1—S2	2.270 (2)	C11A—C12A	1.43 (3)
Zn1—S1	2.271 (2)	C11A—C15A	1.51 (2)
Fe1—C4	2.016 (7)	C11A—H11A	0.98
Fe1—C2	2.027 (9)	C12A—C13A	1.36 (3)
Fe1—C3	2.030 (9)	C12A—H12A	0.98
Fe1—C5	2.033 (9)	C13A—C14A	1.398 (19)
Fe1—C9	2.042 (7)	C13A—H13A	0.98
Fe1—C10	2.046 (7)	C14A—C15A	1.38 (2)
Fe1—C8	2.049 (6)	C14A—H14A	0.9800
Fe1—C1	2.050 (9)	C15A—H15A	0.98
Fe1—C6	2.062 (7)	C11B—C12B	1.43 (3)
Fe1—C7	2.072 (7)	C11B—C15B	1.50 (3)
Fe2—C15A	1.95 (4)	C11B—H11B	0.98
Fe2—C14A	1.99 (6)	C12B—C13B	1.35 (3)
Fe2—C12B	2.00 (3)	C12B—H12B	0.98
Fe2—C13B	2.03 (3)	C13B—C14B	1.40 (2)
Fe2—C20	2.030 (9)	C13B—H13B	0.98
Fe2—C16	2.040 (8)	C14B—C15B	1.39 (2)
Fe2—C11A	2.053 (19)	C14B—H14B	0.98
Fe2—C19	2.054 (9)	C15B—H15B	0.98
Fe2—C11B	2.06 (2)	C16—C17	1.412 (12)
Fe2—C17	2.061 (8)	C16—C20	1.449 (11)
Fe2—C14B	2.07 (7)	C16—H16A	0.98
Fe2—C12A	2.071 (18)	C17—C18	1.421 (13)
S1—C22	1.766 (7)	C17—H17A	0.98
S2—C30	1.771 (7)	C18—C19	1.452 (11)
N1—C21	1.259 (9)	C18—H18A	0.98
N1—N2	1.398 (9)	C19—C20	1.415 (12)
N2—C22	1.301 (9)	C19—H19A	0.98
N3—C22	1.370 (10)	C20—C29	1.444 (10)
N3—C23	1.397 (9)	C21—H21A	0.93
N3—H3B	0.86	C23—C24	1.385 (11)
N4—C29	1.305 (10)	C23—C28	1.394 (11)
N4—N5	1.388 (9)	C24—C25	1.404 (12)
N5—C30	1.308 (10)	C24—H24A	0.93
N6—C30	1.364 (10)	C25—C26	1.388 (12)
N6—C31	1.411 (9)	C25—H25A	0.93
N6—H6B	0.86	C26—C27	1.369 (11)
C1—C2	1.382 (13)	C26—H26A	0.93
C1—C5	1.409 (13)	C27—C28	1.387 (10)
C1—H1A	0.98	C27—H27A	0.93
C2—C3	1.471 (14)	C28—H28A	0.93
C2—H2A	0.98	C29—H29A	0.93
C3—C4	1.353 (15)	C31—C36	1.381 (11)
C3—H3A	0.98	C31—C32	1.386 (11)
C4—C5	1.425 (13)	C32—C33	1.391 (11)
C4—H4A	0.98	C32—H32A	0.93
C5—H5A	0.98	C33—C34	1.383 (12)
C6—C7	1.429 (11)	C33—H33A	0.93
C6—C10	1.444 (11)	C34—C35	1.362 (13)
C6—H6A	0.98	C34—H34A	0.93
C7—C8	1.389 (12)	C35—C36	1.374 (11)
C7—H7A	0.98	C35—H35A	0.93
C8—C9	1.418 (11)	C36—H36A	0.93
C8—H8A	0.98	O1W—H1W1	0.85
C9—C10	1.441 (10)	O1W—H2W1	0.85
			
N4—Zn1—N1	105.0 (2)	C7—C6—H6A	126.0
N4—Zn1—S2	86.35 (19)	C10—C6—H6A	126.0
N1—Zn1—S2	124.5 (2)	Fe1—C6—H6A	126.0
N4—Zn1—S1	124.1 (2)	C8—C7—C6	108.2 (7)
N1—Zn1—S1	87.0 (2)	C8—C7—Fe1	69.4 (4)
S2—Zn1—S1	130.88 (7)	C6—C7—Fe1	69.4 (4)
C4—Fe1—C2	69.2 (4)	C8—C7—H7A	125.9
C4—Fe1—C3	39.1 (4)	C6—C7—H7A	125.9
C2—Fe1—C3	42.5 (4)	Fe1—C7—H7A	125.9
C4—Fe1—C5	41.2 (4)	C7—C8—C9	109.7 (7)
C2—Fe1—C5	68.2 (4)	C7—C8—Fe1	71.2 (4)
C3—Fe1—C5	67.5 (4)	C9—C8—Fe1	69.5 (4)
C4—Fe1—C9	125.1 (4)	C7—C8—H8A	125.2
C2—Fe1—C9	154.8 (4)	C9—C8—H8A	125.2
C3—Fe1—C9	160.9 (4)	Fe1—C8—H8A	125.2
C5—Fe1—C9	107.5 (4)	C8—C9—C10	107.7 (7)
C4—Fe1—C10	105.6 (3)	C8—C9—Fe1	70.0 (4)
C2—Fe1—C10	163.0 (4)	C10—C9—Fe1	69.5 (4)
C3—Fe1—C10	123.5 (3)	C8—C9—H9A	126.2
C5—Fe1—C10	119.1 (4)	C10—C9—H9A	126.2
C9—Fe1—C10	41.3 (3)	Fe1—C9—H9A	126.2
C4—Fe1—C8	163.5 (4)	C9—C10—C6	106.6 (6)
C2—Fe1—C8	120.8 (4)	C9—C10—C21	123.0 (7)
C3—Fe1—C8	156.9 (4)	C6—C10—C21	130.4 (7)
C5—Fe1—C8	126.9 (4)	C9—C10—Fe1	69.2 (4)
C9—Fe1—C8	40.6 (3)	C6—C10—Fe1	70.0 (4)
C10—Fe1—C8	68.6 (3)	C21—C10—Fe1	126.8 (5)
C4—Fe1—C1	68.4 (3)	C12A—C11A—C15A	101.9 (16)
C2—Fe1—C1	39.6 (4)	C12A—C11A—Fe2	70.4 (11)
C3—Fe1—C1	67.9 (4)	C15A—C11A—Fe2	64.1 (15)
C5—Fe1—C1	40.4 (4)	C12A—C11A—H11A	129.0
C9—Fe1—C1	121.2 (3)	C15A—C11A—H11A	129.0
C10—Fe1—C1	155.0 (3)	Fe2—C11A—H11A	129.0
C8—Fe1—C1	110.0 (3)	C13A—C12A—C11A	112.5 (17)
C4—Fe1—C6	119.0 (3)	C13A—C12A—Fe2	71.5 (12)
C2—Fe1—C6	126.1 (3)	C11A—C12A—Fe2	69.0 (11)
C3—Fe1—C6	107.5 (3)	C13A—C12A—H12A	123.7
C5—Fe1—C6	154.5 (4)	C11A—C12A—H12A	123.7
C9—Fe1—C6	68.6 (3)	Fe2—C12A—H12A	123.7
C10—Fe1—C6	41.2 (3)	C12A—C13A—C14A	107.9 (19)
C8—Fe1—C6	67.5 (3)	C12A—C13A—Fe2	70.4 (12)
C1—Fe1—C6	163.2 (3)	C14A—C13A—Fe2	66 (2)
C4—Fe1—C7	154.6 (4)	C12A—C13A—H13A	126.0
C2—Fe1—C7	108.7 (3)	C14A—C13A—H13A	126.0
C3—Fe1—C7	122.1 (4)	Fe2—C13A—H13A	126.0
C5—Fe1—C7	163.4 (3)	C15A—C14A—C13A	109.7 (16)
C9—Fe1—C7	67.8 (3)	C15A—C14A—Fe2	68 (2)
C10—Fe1—C7	68.7 (3)	C13A—C14A—Fe2	74 (2)
C8—Fe1—C7	39.4 (3)	C13A—C14A—H14A	125.2
C1—Fe1—C7	127.1 (3)	Fe2—C14A—H14A	125.2
C6—Fe1—C7	40.5 (3)	C14A—C15A—C11A	107.9 (18)
C15A—Fe2—C14A	41.1 (11)	C14A—C15A—Fe2	71 (3)
C15A—Fe2—C12B	55.9 (10)	C11A—C15A—Fe2	71.7 (14)
C14A—Fe2—C12B	69.3 (15)	C14A—C15A—H15A	126.0
C15A—Fe2—C13B	56.2 (13)	C11A—C15A—H15A	126.0
C14A—Fe2—C13B	39.5 (15)	Fe2—C15A—H15A	126.0
C12B—Fe2—C13B	39.1 (8)	C12B—C11B—C15B	103.8 (18)
C15A—Fe2—C20	118.6 (8)	C12B—C11B—Fe2	66.9 (15)
C14A—Fe2—C20	110.0 (13)	C15B—C11B—Fe2	71.2 (15)
C12B—Fe2—C20	172.8 (9)	C12B—C11B—H11B	128.0
C13B—Fe2—C20	143.9 (7)	C15B—C11B—H11B	128.0
C15A—Fe2—C16	116.3 (9)	Fe2—C11B—H11B	128.0
C14A—Fe2—C16	138.9 (10)	C13B—C12B—C11B	111.4 (18)
C12B—Fe2—C16	134.3 (9)	C13B—C12B—Fe2	71.6 (16)
C13B—Fe2—C16	171.3 (9)	C11B—C12B—Fe2	71.8 (15)
C20—Fe2—C16	41.7 (3)	C13B—C12B—H12B	124.3
C15A—Fe2—C11A	44.2 (8)	C11B—C12B—H12B	124.3
C14A—Fe2—C11A	70.6 (14)	Fe2—C12B—H12B	124.3
C12B—Fe2—C11A	19.1 (8)	C12B—C13B—C14B	108 (2)
C13B—Fe2—C11A	51.5 (11)	C12B—C13B—Fe2	69.3 (16)
C20—Fe2—C11A	153.7 (8)	C14B—C13B—Fe2	72 (3)
C16—Fe2—C11A	120.4 (8)	C12B—C13B—H13B	126.0
C15A—Fe2—C19	145.3 (7)	C14B—C13B—H13B	126.0
C14A—Fe2—C19	110.9 (13)	Fe2—C13B—H13B	126.0
C12B—Fe2—C19	146.7 (9)	C15B—C14B—C13B	110.6 (18)
C13B—Fe2—C19	119.9 (8)	C15B—C14B—Fe2	73 (3)
C20—Fe2—C19	40.5 (4)	C13B—C14B—Fe2	68 (2)
C16—Fe2—C19	68.7 (4)	C15B—C14B—H14B	124.7
C11A—Fe2—C19	165.4 (7)	C13B—C14B—H14B	124.7
C15A—Fe2—C11B	35.7 (11)	Fe2—C14B—H14B	124.7
C14A—Fe2—C11B	73.7 (14)	C14B—C15B—C11B	106.1 (18)
C12B—Fe2—C11B	41.3 (9)	C14B—C15B—Fe2	69 (3)
C13B—Fe2—C11B	68.4 (11)	C11B—C15B—Fe2	66.8 (13)
C20—Fe2—C11B	131.4 (9)	C14B—C15B—H15B	126.9
C16—Fe2—C11B	103.0 (9)	C11B—C15B—H15B	126.9
C11A—Fe2—C11B	22.3 (6)	Fe2—C15B—H15B	126.9
C19—Fe2—C11B	171.4 (9)	C17—C16—C20	107.9 (8)
C15A—Fe2—C17	139.4 (8)	C17—C16—Fe2	70.7 (5)
C14A—Fe2—C17	178.9 (13)	C20—C16—Fe2	68.8 (5)
C12B—Fe2—C17	111.8 (7)	C17—C16—H16A	126.0
C13B—Fe2—C17	141.5 (8)	C20—C16—H16A	126.0
C20—Fe2—C17	68.9 (3)	Fe2—C16—H16A	126.0
C16—Fe2—C17	40.3 (3)	C16—C17—C18	109.2 (7)
C11A—Fe2—C17	110.5 (7)	C16—C17—Fe2	69.1 (5)
C19—Fe2—C17	68.2 (4)	C18—C17—Fe2	70.5 (5)
C11B—Fe2—C17	107.1 (7)	C16—C17—H17A	125.4
C15A—Fe2—C14B	35.0 (15)	C18—C17—H17A	125.4
C14A—Fe2—C14B	6(2)	Fe2—C17—H17A	125.4
C12B—Fe2—C14B	66.3 (15)	C17—C18—C19	106.9 (7)
C13B—Fe2—C14B	40.0 (11)	C17—C18—Fe2	69.3 (5)
C20—Fe2—C14B	112.3 (14)	C19—C18—Fe2	68.6 (5)
C16—Fe2—C14B	137.0 (11)	C17—C18—H18A	126.5
C11A—Fe2—C14B	65.9 (16)	C19—C18—H18A	126.5
C19—Fe2—C14B	116.4 (14)	Fe2—C18—H18A	126.5
C11B—Fe2—C14B	67.8 (14)	C20—C19—C18	108.5 (8)
C17—Fe2—C14B	174.4 (11)	C20—C19—Fe2	68.8 (5)
C15A—Fe2—C12A	69.1 (9)	C18—C19—Fe2	70.3 (5)
C14A—Fe2—C12A	66.5 (13)	C20—C19—H19A	125.8
C12B—Fe2—C12A	21.7 (6)	C18—C19—H19A	125.8
C13B—Fe2—C12A	28.0 (10)	Fe2—C19—H19A	125.8
C20—Fe2—C12A	165.2 (8)	C19—C20—C29	131.0 (7)
C16—Fe2—C12A	149.1 (8)	C19—C20—C16	107.5 (7)
C11A—Fe2—C12A	40.5 (8)	C29—C20—C16	121.5 (7)
C19—Fe2—C12A	125.7 (7)	C19—C20—Fe2	70.6 (5)
C11B—Fe2—C12A	62.6 (10)	C29—C20—Fe2	123.1 (6)
C17—Fe2—C12A	114.5 (7)	C16—C20—Fe2	69.5 (5)
C14B—Fe2—C12A	65.9 (15)	N1—C21—C10	133.2 (7)
C22—S1—Zn1	92.5 (3)	N1—C21—H21A	113.4
C30—S2—Zn1	92.4 (3)	C10—C21—H21A	113.4
C21—N1—N2	116.6 (6)	N2—C22—N3	118.4 (6)
C21—N1—Zn1	123.7 (5)	N2—C22—S1	128.8 (6)
N2—N1—Zn1	117.9 (4)	N3—C22—S1	112.8 (5)
C22—N2—N1	113.6 (6)	C24—C23—C28	118.4 (8)
C22—N3—C23	130.3 (6)	C24—C23—N3	118.9 (8)
C22—N3—H3B	114.8	C28—C23—N3	122.5 (7)
C23—N3—H3B	114.8	C23—C24—C25	120.9 (9)
C29—N4—N5	117.0 (6)	C23—C24—H24A	119.6
C29—N4—Zn1	124.9 (5)	C25—C24—H24A	119.6
N5—N4—Zn1	117.9 (5)	C26—C25—C24	119.8 (7)
C30—N5—N4	113.8 (6)	C26—C25—H25A	120.1
C30—N6—C31	127.8 (7)	C24—C25—H25A	120.1
C30—N6—H6B	116.1	C27—C26—C25	119.1 (8)
C31—N6—H6B	116.1	C27—C26—H26A	120.4
C2—C1—C5	109.4 (9)	C25—C26—H26A	120.4
C2—C1—Fe1	69.3 (5)	C26—C27—C28	121.5 (8)
C5—C1—Fe1	69.2 (5)	C26—C27—H27A	119.3
C2—C1—H1A	125.3	C28—C27—H27A	119.3
C5—C1—H1A	125.3	C27—C28—C23	120.3 (7)
Fe1—C1—H1A	125.3	C27—C28—H28A	119.9
C1—C2—C3	106.0 (9)	C23—C28—H28A	119.9
C1—C2—Fe1	71.1 (6)	N4—C29—C20	130.4 (7)
C3—C2—Fe1	68.9 (5)	N4—C29—H29A	114.8
C1—C2—H2A	127.0	C20—C29—H29A	114.8
C3—C2—H2A	127.0	N5—C30—N6	118.5 (6)
Fe1—C2—H2A	127.0	N5—C30—S2	127.4 (6)
C4—C3—C2	108.6 (8)	N6—C30—S2	114.0 (5)
C4—C3—Fe1	69.9 (5)	C36—C31—C32	119.2 (8)
C2—C3—Fe1	68.6 (5)	C36—C31—N6	117.1 (8)
C4—C3—H3A	125.7	C32—C31—N6	123.6 (7)
C2—C3—H3A	125.7	C31—C32—C33	120.3 (7)
Fe1—C3—H3A	125.7	C31—C32—H32A	119.9
C3—C4—C5	108.6 (7)	C33—C32—H32A	119.9
C3—C4—Fe1	71.0 (5)	C34—C33—C32	119.5 (9)
C5—C4—Fe1	70.1 (4)	C34—C33—H33A	120.2
C3—C4—H4A	125.7	C32—C33—H33A	120.2
C5—C4—H4A	125.7	C35—C34—C33	119.7 (8)
Fe1—C4—H4A	125.7	C35—C34—H34A	120.2
C1—C5—C4	107.4 (8)	C33—C34—H34A	120.2
C1—C5—Fe1	70.5 (5)	C34—C35—C36	121.4 (8)
C4—C5—Fe1	68.7 (5)	C34—C35—H35A	119.3
C1—C5—H5A	126.3	C36—C35—H35A	119.3
C4—C5—H5A	126.3	C35—C36—C31	119.8 (8)
Fe1—C5—H5A	126.3	C35—C36—H36A	120.1
C7—C6—C10	107.9 (7)	C31—C36—H36A	120.1
C7—C6—Fe1	70.1 (4)	H1W1—O1W—H2W1	107.7
C10—C6—Fe1	68.8 (4)		
			
N4—Zn1—S1—C22	108.9 (3)	C20—Fe2—C14A—C13A	129.2 (17)
N1—Zn1—S1—C22	2.8 (3)	C16—Fe2—C14A—C13A	166.9 (13)
S2—Zn1—S1—C22	−132.1 (2)	C11A—Fe2—C14A—C13A	−79 (2)
N4—Zn1—S2—C30	10.8 (3)	C19—Fe2—C14A—C13A	86 (2)
N1—Zn1—S2—C30	116.5 (3)	C11B—Fe2—C14A—C13A	−102 (2)
S1—Zn1—S2—C30	−122.8 (3)	C14B—Fe2—C14A—C13A	−118 (22)
N4—Zn1—N1—C21	38.0 (7)	C12A—Fe2—C14A—C13A	−35.3 (16)
S2—Zn1—N1—C21	−58.0 (6)	C13A—C14A—C15A—C11A	−1(5)
S1—Zn1—N1—C21	162.6 (6)	Fe2—C14A—C15A—C11A	−63 (2)
N4—Zn1—N1—N2	−126.5 (5)	C13A—C14A—C15A—Fe2	62 (4)
S2—Zn1—N1—N2	137.5 (4)	C12A—C11A—C15A—C14A	1(4)
S1—Zn1—N1—N2	−1.9 (4)	Fe2—C11A—C15A—C14A	62 (4)
C21—N1—N2—C22	−166.1 (6)	C12A—C11A—C15A—Fe2	−61.1 (15)
Zn1—N1—N2—C22	−0.5 (7)	C12B—Fe2—C15A—C14A	−97 (2)
N1—Zn1—N4—C29	37.5 (8)	C13B—Fe2—C15A—C14A	−50 (2)
S2—Zn1—N4—C29	162.3 (7)	C20—Fe2—C15A—C14A	88.2 (18)
S1—Zn1—N4—C29	−59.2 (7)	C16—Fe2—C15A—C14A	135.4 (17)
N1—Zn1—N4—N5	−137.7 (5)	C11A—Fe2—C15A—C14A	−117 (2)
S2—Zn1—N4—N5	−12.9 (5)	C19—Fe2—C15A—C14A	45 (2)
S1—Zn1—N4—N5	125.6 (5)	C11B—Fe2—C15A—C14A	−150 (3)
C29—N4—N5—C30	−167.1 (7)	C17—Fe2—C15A—C14A	178.4 (17)
Zn1—N4—N5—C30	8.5 (8)	C14B—Fe2—C15A—C14A	0(4)
C4—Fe1—C1—C2	83.1 (7)	C12A—Fe2—C15A—C14A	−77.8 (19)
C3—Fe1—C1—C2	40.8 (6)	C14A—Fe2—C15A—C11A	117 (2)
C5—Fe1—C1—C2	121.6 (8)	C12B—Fe2—C15A—C11A	19.7 (14)
C9—Fe1—C1—C2	−158.1 (6)	C13B—Fe2—C15A—C11A	67.3 (16)
C10—Fe1—C1—C2	163.4 (7)	C20—Fe2—C15A—C11A	−154.8 (12)
C8—Fe1—C1—C2	−114.5 (6)	C16—Fe2—C15A—C11A	−107.5 (13)
C6—Fe1—C1—C2	−36.3 (14)	C19—Fe2—C15A—C11A	162.4 (13)
C7—Fe1—C1—C2	−73.6 (7)	C11B—Fe2—C15A—C11A	−32.5 (13)
C4—Fe1—C1—C5	−38.5 (6)	C17—Fe2—C15A—C11A	−64.5 (18)
C2—Fe1—C1—C5	−121.6 (8)	C14B—Fe2—C15A—C11A	117 (3)
C3—Fe1—C1—C5	−80.7 (6)	C12A—Fe2—C15A—C11A	39.2 (13)
C9—Fe1—C1—C5	80.4 (6)	C15A—Fe2—C11B—C12B	97 (2)
C10—Fe1—C1—C5	41.8 (9)	C14A—Fe2—C11B—C12B	77.0 (18)
C8—Fe1—C1—C5	123.9 (5)	C13B—Fe2—C11B—C12B	35.6 (12)
C6—Fe1—C1—C5	−157.9 (10)	C20—Fe2—C11B—C12B	179.6 (11)
C7—Fe1—C1—C5	164.9 (5)	C16—Fe2—C11B—C12B	−145.4 (13)
C5—C1—C2—C3	−2.8 (10)	C11A—Fe2—C11B—C12B	−1(2)
Fe1—C1—C2—C3	−60.4 (6)	C17—Fe2—C11B—C12B	−103.8 (14)
C5—C1—C2—Fe1	57.6 (7)	C14B—Fe2—C11B—C12B	78.9 (16)
C4—Fe1—C2—C1	−80.8 (6)	C12A—Fe2—C11B—C12B	5.4 (13)
C3—Fe1—C2—C1	−116.4 (8)	C15A—Fe2—C11B—C15B	−17 (2)
C5—Fe1—C2—C1	−36.5 (5)	C14A—Fe2—C11B—C15B	−37.7 (19)
C9—Fe1—C2—C1	48.6 (11)	C12B—Fe2—C11B—C15B	−115 (2)
C10—Fe1—C2—C1	−155.7 (10)	C13B—Fe2—C11B—C15B	−79.2 (15)
C8—Fe1—C2—C1	84.5 (6)	C20—Fe2—C11B—C15B	64.8 (17)
C6—Fe1—C2—C1	167.8 (5)	C16—Fe2—C11B—C15B	99.9 (15)
C7—Fe1—C2—C1	126.1 (5)	C11A—Fe2—C11B—C15B	−116 (3)
C4—Fe1—C2—C3	35.5 (6)	C17—Fe2—C11B—C15B	141.4 (14)
C5—Fe1—C2—C3	79.9 (6)	C14B—Fe2—C11B—C15B	−35.9 (16)
C9—Fe1—C2—C3	165.0 (8)	C12A—Fe2—C11B—C15B	−109.4 (17)
C10—Fe1—C2—C3	−39.3 (14)	C15B—C11B—C12B—C13B	1(3)
C8—Fe1—C2—C3	−159.1 (5)	Fe2—C11B—C12B—C13B	−61 (2)
C1—Fe1—C2—C3	116.4 (8)	C15B—C11B—C12B—Fe2	62 (2)
C6—Fe1—C2—C3	−75.9 (6)	C15A—Fe2—C12B—C13B	76.6 (19)
C7—Fe1—C2—C3	−117.5 (6)	C14A—Fe2—C12B—C13B	32 (2)
C1—C2—C3—C4	3.3 (10)	C16—Fe2—C12B—C13B	171.7 (14)
Fe1—C2—C3—C4	−58.6 (6)	C11A—Fe2—C12B—C13B	123 (4)
C1—C2—C3—Fe1	61.9 (6)	C19—Fe2—C12B—C13B	−64 (2)
C2—Fe1—C3—C4	120.5 (7)	C11B—Fe2—C12B—C13B	121 (2)
C5—Fe1—C3—C4	38.7 (5)	C17—Fe2—C12B—C13B	−147.6 (15)
C9—Fe1—C3—C4	−39.8 (13)	C14B—Fe2—C12B—C13B	38.2 (14)
C10—Fe1—C3—C4	−72.3 (6)	C12A—Fe2—C12B—C13B	−46 (3)
C8—Fe1—C3—C4	171.8 (7)	C15A—Fe2—C12B—C11B	−44.4 (18)
C1—Fe1—C3—C4	82.5 (6)	C14A—Fe2—C12B—C11B	−88.5 (18)
C6—Fe1—C3—C4	−114.8 (5)	C13B—Fe2—C12B—C11B	−121 (2)
C7—Fe1—C3—C4	−156.7 (5)	C16—Fe2—C12B—C11B	50.7 (18)
C4—Fe1—C3—C2	−120.5 (7)	C11A—Fe2—C12B—C11B	2(3)
C5—Fe1—C3—C2	−81.9 (6)	C19—Fe2—C12B—C11B	174.6 (12)
C9—Fe1—C3—C2	−160.3 (9)	C17—Fe2—C12B—C11B	91.4 (14)
C10—Fe1—C3—C2	167.2 (5)	C14B—Fe2—C12B—C11B	−82.8 (14)
C8—Fe1—C3—C2	51.3 (10)	C12A—Fe2—C12B—C11B	−167 (3)
C1—Fe1—C3—C2	−38.1 (5)	C11B—C12B—C13B—C14B	−1(5)
C6—Fe1—C3—C2	124.7 (5)	Fe2—C12B—C13B—C14B	−62 (4)
C7—Fe1—C3—C2	82.8 (6)	C11B—C12B—C13B—Fe2	61 (2)
C2—C3—C4—C5	−2.5 (10)	C15A—Fe2—C13B—C12B	−75.7 (19)
Fe1—C3—C4—C5	−60.3 (6)	C14A—Fe2—C13B—C12B	−128 (3)
C2—C3—C4—Fe1	57.8 (6)	C20—Fe2—C13B—C12B	−169.2 (14)
C2—Fe1—C4—C3	−38.5 (5)	C11A—Fe2—C13B—C12B	−20.5 (14)
C5—Fe1—C4—C3	−118.9 (7)	C19—Fe2—C13B—C12B	145.2 (15)
C9—Fe1—C4—C3	165.2 (5)	C11B—Fe2—C13B—C12B	−37.5 (15)
C10—Fe1—C4—C3	124.4 (5)	C17—Fe2—C13B—C12B	53 (2)
C8—Fe1—C4—C3	−168.6 (10)	C14B—Fe2—C13B—C12B	−118 (3)
C1—Fe1—C4—C3	−81.2 (6)	C12A—Fe2—C13B—C12B	34.5 (15)
C6—Fe1—C4—C3	82.1 (6)	C15A—Fe2—C13B—C14B	43 (2)
C7—Fe1—C4—C3	51.4 (10)	C14A—Fe2—C13B—C14B	−9(3)
C2—Fe1—C4—C5	80.4 (6)	C12B—Fe2—C13B—C14B	118 (3)
C3—Fe1—C4—C5	118.9 (7)	C20—Fe2—C13B—C14B	−51 (2)
C9—Fe1—C4—C5	−76.0 (6)	C11A—Fe2—C13B—C14B	98 (2)
C10—Fe1—C4—C5	−116.7 (6)	C19—Fe2—C13B—C14B	−97 (2)
C8—Fe1—C4—C5	−49.8 (13)	C11B—Fe2—C13B—C14B	81 (2)
C1—Fe1—C4—C5	37.7 (6)	C17—Fe2—C13B—C14B	171 (2)
C6—Fe1—C4—C5	−159.0 (5)	C12A—Fe2—C13B—C14B	153 (3)
C7—Fe1—C4—C5	170.3 (7)	C12B—C13B—C14B—C15B	−1(7)
C2—C1—C5—C4	1.4 (10)	Fe2—C13B—C14B—C15B	−61 (5)
Fe1—C1—C5—C4	59.0 (6)	C12B—C13B—C14B—Fe2	60 (2)
C2—C1—C5—Fe1	−57.6 (7)	C15A—Fe2—C14B—C15B	20.1 (19)
C3—C4—C5—C1	0.7 (10)	C14A—Fe2—C14B—C15B	−158 (23)
Fe1—C4—C5—C1	−60.1 (6)	C12B—Fe2—C14B—C15B	84 (2)
C3—C4—C5—Fe1	60.9 (6)	C13B—Fe2—C14B—C15B	121 (3)
C4—Fe1—C5—C1	118.6 (8)	C20—Fe2—C14B—C15B	−88 (2)
C2—Fe1—C5—C1	35.8 (6)	C16—Fe2—C14B—C15B	−46 (3)
C3—Fe1—C5—C1	81.9 (6)	C11A—Fe2—C14B—C15B	63 (2)
C9—Fe1—C5—C1	−117.8 (5)	C19—Fe2—C14B—C15B	−133 (2)
C10—Fe1—C5—C1	−161.2 (5)	C11B—Fe2—C14B—C15B	39 (2)
C8—Fe1—C5—C1	−77.1 (6)	C12A—Fe2—C14B—C15B	108 (3)
C6—Fe1—C5—C1	165.3 (7)	C15A—Fe2—C14B—C13B	−101 (3)
C7—Fe1—C5—C1	−46.7 (15)	C14A—Fe2—C14B—C13B	81 (21)
C2—Fe1—C5—C4	−82.8 (6)	C12B—Fe2—C14B—C13B	−37.3 (18)
C3—Fe1—C5—C4	−36.7 (5)	C20—Fe2—C14B—C13B	150.4 (16)
C9—Fe1—C5—C4	123.6 (6)	C16—Fe2—C14B—C13B	−167.7 (13)
C10—Fe1—C5—C4	80.2 (6)	C11A—Fe2—C14B—C13B	−58.2 (19)
C8—Fe1—C5—C4	164.3 (5)	C19—Fe2—C14B—C13B	105.9 (19)
C1—Fe1—C5—C4	−118.6 (8)	C11B—Fe2—C14B—C13B	−82 (2)
C6—Fe1—C5—C4	46.7 (11)	C12A—Fe2—C14B—C13B	−13.6 (16)
C7—Fe1—C5—C4	−165.3 (11)	C13B—C14B—C15B—C11B	2(7)
C4—Fe1—C6—C7	−160.3 (5)	Fe2—C14B—C15B—C11B	−56 (3)
C2—Fe1—C6—C7	−76.0 (6)	C13B—C14B—C15B—Fe2	58 (4)
C3—Fe1—C6—C7	−119.4 (5)	C12B—C11B—C15B—C14B	−2(5)
C5—Fe1—C6—C7	166.5 (8)	Fe2—C11B—C15B—C14B	58 (4)
C9—Fe1—C6—C7	80.5 (5)	C12B—C11B—C15B—Fe2	−59.4 (18)
C10—Fe1—C6—C7	119.4 (6)	C15A—Fe2—C15B—C14B	−65 (6)
C8—Fe1—C6—C7	36.6 (4)	C14A—Fe2—C15B—C14B	3(4)
C1—Fe1—C6—C7	−48.1 (13)	C12B—Fe2—C15B—C14B	−79 (3)
C4—Fe1—C6—C10	80.3 (5)	C13B—Fe2—C15B—C14B	−37 (2)
C2—Fe1—C6—C10	164.6 (5)	C20—Fe2—C15B—C14B	106 (2)
C3—Fe1—C6—C10	121.2 (5)	C16—Fe2—C15B—C14B	149 (2)
C5—Fe1—C6—C10	47.1 (10)	C11A—Fe2—C15B—C14B	−95 (3)
C9—Fe1—C6—C10	−38.9 (4)	C19—Fe2—C15B—C14B	73 (3)
C8—Fe1—C6—C10	−82.8 (4)	C11B—Fe2—C15B—C14B	−119 (3)
C1—Fe1—C6—C10	−167.5 (10)	C17—Fe2—C15B—C14B	−178 (2)
C7—Fe1—C6—C10	−119.4 (6)	C12A—Fe2—C15B—C14B	−62 (3)
C10—C6—C7—C8	−0.2 (8)	C15A—Fe2—C15B—C11B	54 (6)
Fe1—C6—C7—C8	−58.8 (5)	C14A—Fe2—C15B—C11B	123 (3)
C10—C6—C7—Fe1	58.6 (5)	C12B—Fe2—C15B—C11B	40.3 (14)
C4—Fe1—C7—C8	163.3 (7)	C13B—Fe2—C15B—C11B	82.7 (16)
C2—Fe1—C7—C8	−116.1 (5)	C20—Fe2—C15B—C11B	−135.1 (14)
C3—Fe1—C7—C8	−161.1 (5)	C16—Fe2—C15B—C11B	−91.5 (15)
C5—Fe1—C7—C8	−39.6 (15)	C11A—Fe2—C15B—C11B	24.6 (12)
C9—Fe1—C7—C8	37.2 (4)	C19—Fe2—C15B—C11B	−167.4 (13)
C10—Fe1—C7—C8	81.8 (5)	C17—Fe2—C15B—C11B	−59 (2)
C1—Fe1—C7—C8	−75.9 (6)	C14B—Fe2—C15B—C11B	119 (3)
C6—Fe1—C7—C8	119.8 (6)	C12A—Fe2—C15B—C11B	57.9 (16)
C4—Fe1—C7—C6	43.5 (10)	C15A—Fe2—C16—C17	136.6 (9)
C2—Fe1—C7—C6	124.1 (5)	C14A—Fe2—C16—C17	−178.8 (18)
C3—Fe1—C7—C6	79.0 (6)	C12B—Fe2—C16—C17	69.6 (13)
C5—Fe1—C7—C6	−159.5 (12)	C20—Fe2—C16—C17	−119.1 (9)
C9—Fe1—C7—C6	−82.6 (5)	C11A—Fe2—C16—C17	86.2 (9)
C10—Fe1—C7—C6	−38.0 (4)	C19—Fe2—C16—C17	−81.1 (6)
C8—Fe1—C7—C6	−119.8 (6)	C11B—Fe2—C16—C17	101.2 (10)
C1—Fe1—C7—C6	164.3 (5)	C14B—Fe2—C16—C17	173 (2)
C6—C7—C8—C9	−0.2 (8)	C12A—Fe2—C16—C17	43.6 (15)
Fe1—C7—C8—C9	−59.0 (5)	C15A—Fe2—C16—C20	−104.3 (9)
C6—C7—C8—Fe1	58.8 (5)	C14A—Fe2—C16—C20	−59.7 (19)
C4—Fe1—C8—C7	−154.2 (10)	C12B—Fe2—C16—C20	−171.3 (12)
C2—Fe1—C8—C7	82.1 (5)	C11A—Fe2—C16—C20	−154.7 (8)
C3—Fe1—C8—C7	44.2 (10)	C19—Fe2—C16—C20	38.0 (5)
C5—Fe1—C8—C7	166.8 (5)	C11B—Fe2—C16—C20	−139.7 (9)
C9—Fe1—C8—C7	−120.5 (6)	C17—Fe2—C16—C20	119.1 (9)
C10—Fe1—C8—C7	−82.1 (5)	C14B—Fe2—C16—C20	−68 (2)
C1—Fe1—C8—C7	124.6 (5)	C12A—Fe2—C16—C20	162.7 (13)
C6—Fe1—C8—C7	−37.6 (5)	C20—C16—C17—C18	0.4 (11)
C4—Fe1—C8—C9	−33.7 (13)	Fe2—C16—C17—C18	59.2 (7)
C2—Fe1—C8—C9	−157.4 (5)	C20—C16—C17—Fe2	−58.9 (6)
C3—Fe1—C8—C9	164.7 (8)	C15A—Fe2—C17—C16	−71.1 (13)
C5—Fe1—C8—C9	−72.7 (6)	C12B—Fe2—C17—C16	−133.7 (11)
C10—Fe1—C8—C9	38.4 (5)	C13B—Fe2—C17—C16	−166.6 (13)
C1—Fe1—C8—C9	−114.9 (5)	C20—Fe2—C17—C16	38.6 (6)
C6—Fe1—C8—C9	82.9 (5)	C11A—Fe2—C17—C16	−113.3 (9)
C7—Fe1—C8—C9	120.5 (6)	C19—Fe2—C17—C16	82.3 (6)
C7—C8—C9—C10	0.5 (8)	C11B—Fe2—C17—C16	−90.0 (11)
Fe1—C8—C9—C10	−59.5 (5)	C12A—Fe2—C17—C16	−157.1 (9)
C7—C8—C9—Fe1	60.0 (5)	C15A—Fe2—C17—C18	168.2 (12)
C4—Fe1—C9—C8	168.9 (5)	C12B—Fe2—C17—C18	105.7 (11)
C2—Fe1—C9—C8	50.7 (10)	C13B—Fe2—C17—C18	72.8 (14)
C3—Fe1—C9—C8	−161.6 (10)	C20—Fe2—C17—C18	−82.1 (5)
C5—Fe1—C9—C8	126.8 (5)	C16—Fe2—C17—C18	−120.6 (8)
C10—Fe1—C9—C8	−118.7 (7)	C11A—Fe2—C17—C18	126.1 (9)
C1—Fe1—C9—C8	84.7 (5)	C19—Fe2—C17—C18	−38.4 (5)
C6—Fe1—C9—C8	−79.9 (5)	C11B—Fe2—C17—C18	149.4 (10)
C7—Fe1—C9—C8	−36.2 (5)	C12A—Fe2—C17—C18	82.3 (9)
C4—Fe1—C9—C10	−72.4 (6)	C16—C17—C18—C19	0.1 (11)
C2—Fe1—C9—C10	169.5 (7)	Fe2—C17—C18—C19	58.5 (6)
C3—Fe1—C9—C10	−42.8 (13)	C16—C17—C18—Fe2	−58.4 (7)
C5—Fe1—C9—C10	−114.5 (5)	C14A—Fe2—C18—C17	178.3 (19)
C8—Fe1—C9—C10	118.7 (7)	C12B—Fe2—C18—C17	−93.1 (10)
C1—Fe1—C9—C10	−156.5 (4)	C13B—Fe2—C18—C17	−137.4 (9)
C6—Fe1—C9—C10	38.8 (5)	C20—Fe2—C18—C17	81.7 (5)
C7—Fe1—C9—C10	82.5 (5)	C16—Fe2—C18—C17	36.8 (5)
C8—C9—C10—C6	−0.6 (8)	C11A—Fe2—C18—C17	−75.5 (11)
Fe1—C9—C10—C6	−60.4 (5)	C19—Fe2—C18—C17	118.8 (7)
C8—C9—C10—C21	−179.0 (6)	C11B—Fe2—C18—C17	−48.0 (15)
Fe1—C9—C10—C21	121.2 (7)	C14B—Fe2—C18—C17	−179 (2)
C8—C9—C10—Fe1	59.8 (5)	C12A—Fe2—C18—C17	−111.8 (9)
C7—C6—C10—C9	0.4 (8)	C14A—Fe2—C18—C19	59 (2)
Fe1—C6—C10—C9	59.9 (5)	C12B—Fe2—C18—C19	148.1 (10)
C7—C6—C10—C21	178.7 (7)	C13B—Fe2—C18—C19	103.8 (9)
Fe1—C6—C10—C21	−121.9 (7)	C20—Fe2—C18—C19	−37.1 (5)
C7—C6—C10—Fe1	−59.4 (5)	C16—Fe2—C18—C19	−82.1 (6)
C4—Fe1—C10—C9	125.9 (5)	C11A—Fe2—C18—C19	165.7 (11)
C2—Fe1—C10—C9	−164.6 (11)	C11B—Fe2—C18—C19	−166.8 (14)
C3—Fe1—C10—C9	164.5 (5)	C17—Fe2—C18—C19	−118.8 (7)
C5—Fe1—C10—C9	83.6 (5)	C14B—Fe2—C18—C19	62 (2)
C8—Fe1—C10—C9	−37.8 (5)	C12A—Fe2—C18—C19	129.3 (9)
C1—Fe1—C10—C9	53.9 (8)	C17—C18—C19—C20	−0.5 (10)
C6—Fe1—C10—C9	−117.6 (6)	Fe2—C18—C19—C20	58.4 (6)
C7—Fe1—C10—C9	−80.2 (5)	C17—C18—C19—Fe2	−58.9 (6)
C4—Fe1—C10—C6	−116.5 (5)	C15A—Fe2—C19—C20	66.8 (17)
C2—Fe1—C10—C6	−47.1 (13)	C14A—Fe2—C19—C20	96.8 (10)
C3—Fe1—C10—C6	−77.9 (6)	C12B—Fe2—C19—C20	−179.4 (13)
C5—Fe1—C10—C6	−158.9 (5)	C13B—Fe2—C19—C20	139.5 (10)
C9—Fe1—C10—C6	117.6 (6)	C16—Fe2—C19—C20	−39.1 (5)
C8—Fe1—C10—C6	79.8 (5)	C11A—Fe2—C19—C20	−170 (3)
C1—Fe1—C10—C6	171.5 (7)	C17—Fe2—C19—C20	−82.5 (5)
C7—Fe1—C10—C6	37.4 (4)	C14B—Fe2—C19—C20	94.0 (12)
C4—Fe1—C10—C21	9.7 (8)	C12A—Fe2—C19—C20	172.3 (9)
C2—Fe1—C10—C21	79.1 (14)	C15A—Fe2—C19—C18	−173.3 (16)
C3—Fe1—C10—C21	48.2 (9)	C14A—Fe2—C19—C18	−143.3 (11)
C5—Fe1—C10—C21	−32.7 (8)	C12B—Fe2—C19—C18	−59.5 (15)
C9—Fe1—C10—C21	−116.3 (9)	C13B—Fe2—C19—C18	−100.5 (10)
C8—Fe1—C10—C21	−154.0 (8)	C20—Fe2—C19—C18	120.0 (8)
C1—Fe1—C10—C21	−62.4 (10)	C16—Fe2—C19—C18	80.9 (6)
C6—Fe1—C10—C21	126.2 (8)	C11A—Fe2—C19—C18	−50 (3)
C7—Fe1—C10—C21	163.5 (8)	C17—Fe2—C19—C18	37.5 (5)
C15A—Fe2—C11A—C12A	114.6 (19)	C14B—Fe2—C19—C18	−146.0 (12)
C14A—Fe2—C11A—C12A	76.3 (13)	C12A—Fe2—C19—C18	−67.8 (10)
C12B—Fe2—C11A—C12A	−6(3)	C18—C19—C20—C29	−176.8 (9)
C13B—Fe2—C11A—C12A	36.2 (17)	Fe2—C19—C20—C29	−117.4 (10)
C20—Fe2—C11A—C12A	172.3 (12)	C18—C19—C20—C16	0.8 (10)
C16—Fe2—C11A—C12A	−147.7 (12)	Fe2—C19—C20—C16	60.1 (6)
C19—Fe2—C11A—C12A	−22 (4)	C18—C19—C20—Fe2	−59.3 (6)
C11B—Fe2—C11A—C12A	171 (3)	C17—C16—C20—C19	−0.7 (11)
C17—Fe2—C11A—C12A	−104.2 (13)	Fe2—C16—C20—C19	−60.8 (6)
C14B—Fe2—C11A—C12A	80.5 (18)	C17—C16—C20—C29	177.1 (8)
C14A—Fe2—C11A—C15A	−38.3 (12)	Fe2—C16—C20—C29	117.0 (8)
C12B—Fe2—C11A—C15A	−121 (4)	C17—C16—C20—Fe2	60.1 (7)
C13B—Fe2—C11A—C15A	−78.4 (18)	C15A—Fe2—C20—C19	−143.4 (10)
C20—Fe2—C11A—C15A	58 (2)	C14A—Fe2—C20—C19	−99.1 (10)
C16—Fe2—C11A—C15A	97.7 (13)	C13B—Fe2—C20—C19	−72.5 (16)
C19—Fe2—C11A—C15A	−137 (3)	C16—Fe2—C20—C19	118.1 (7)
C11B—Fe2—C11A—C15A	56 (3)	C11A—Fe2—C20—C19	174.4 (15)
C17—Fe2—C11A—C15A	141.2 (13)	C11B—Fe2—C20—C19	175.4 (10)
C14B—Fe2—C11A—C15A	−34.1 (19)	C17—Fe2—C20—C19	80.8 (6)
C12A—Fe2—C11A—C15A	−114.6 (19)	C14B—Fe2—C20—C19	−105.1 (11)
C15A—C11A—C12A—C13A	−1(3)	C12A—Fe2—C20—C19	−25 (3)
Fe2—C11A—C12A—C13A	−58.2 (17)	C15A—Fe2—C20—C29	−16.5 (12)
C15A—C11A—C12A—Fe2	56.7 (18)	C14A—Fe2—C20—C29	27.8 (11)
C15A—Fe2—C12A—C13A	81.4 (13)	C13B—Fe2—C20—C29	54.4 (17)
C14A—Fe2—C12A—C13A	37.0 (14)	C16—Fe2—C20—C29	−115.0 (9)
C12B—Fe2—C12A—C13A	130 (3)	C11A—Fe2—C20—C29	−58.6 (17)
C13B—Fe2—C12A—C13A	25 (2)	C19—Fe2—C20—C29	126.9 (9)
C20—Fe2—C12A—C13A	−43 (3)	C11B—Fe2—C20—C29	−57.7 (12)
C16—Fe2—C12A—C13A	−171.9 (14)	C17—Fe2—C20—C29	−152.3 (8)
C11A—Fe2—C12A—C13A	124.1 (18)	C14B—Fe2—C20—C29	21.8 (12)
C19—Fe2—C12A—C13A	−62.6 (14)	C12A—Fe2—C20—C29	102 (3)
C11B—Fe2—C12A—C13A	120.1 (17)	C15A—Fe2—C20—C16	98.5 (10)
C17—Fe2—C12A—C13A	−142.6 (12)	C14A—Fe2—C20—C16	142.8 (10)
C14B—Fe2—C12A—C13A	43.6 (19)	C13B—Fe2—C20—C16	169.4 (15)
C15A—Fe2—C12A—C11A	−42.7 (12)	C11A—Fe2—C20—C16	56.4 (16)
C14A—Fe2—C12A—C11A	−87.1 (15)	C19—Fe2—C20—C16	−118.1 (7)
C12B—Fe2—C12A—C11A	6(2)	C11B—Fe2—C20—C16	57.3 (11)
C13B—Fe2—C12A—C11A	−99 (3)	C17—Fe2—C20—C16	−37.3 (6)
C20—Fe2—C12A—C11A	−166.7 (19)	C14B—Fe2—C20—C16	136.8 (11)
C16—Fe2—C12A—C11A	64.0 (19)	C12A—Fe2—C20—C16	−143 (2)
C19—Fe2—C12A—C11A	173.3 (11)	N2—N1—C21—C10	3.1 (11)
C11B—Fe2—C12A—C11A	−4.0 (13)	Zn1—N1—C21—C10	−161.6 (6)
C17—Fe2—C12A—C11A	93.3 (13)	C9—C10—C21—N1	−176.8 (8)
C14B—Fe2—C12A—C11A	−80.5 (19)	C6—C10—C21—N1	5.2 (13)
C11A—C12A—C13A—C14A	1(4)	Fe1—C10—C21—N1	−89.2 (10)
Fe2—C12A—C13A—C14A	−56 (3)	N1—N2—C22—N3	−177.5 (6)
C11A—C12A—C13A—Fe2	56.8 (16)	N1—N2—C22—S1	4.1 (9)
C15A—Fe2—C13A—C12A	−83.2 (14)	C23—N3—C22—N2	11.5 (11)
C14A—Fe2—C13A—C12A	−121 (3)	C23—N3—C22—S1	−169.8 (6)
C12B—Fe2—C13A—C12A	−20.5 (11)	Zn1—S1—C22—N2	−4.8 (7)
C13B—Fe2—C13A—C12A	−43 (4)	Zn1—S1—C22—N3	176.7 (5)
C20—Fe2—C13A—C12A	166.9 (10)	C22—N3—C23—C24	−161.8 (8)
C11A—Fe2—C13A—C12A	−35.5 (12)	C22—N3—C23—C28	23.6 (11)
C19—Fe2—C13A—C12A	132.2 (12)	C28—C23—C24—C25	0.1 (13)
C11B—Fe2—C13A—C12A	−50.5 (14)	N3—C23—C24—C25	−174.7 (7)
C17—Fe2—C13A—C12A	60.5 (19)	C23—C24—C25—C26	1.2 (13)
C14B—Fe2—C13A—C12A	−113 (3)	C24—C25—C26—C27	−1.8 (12)
C15A—Fe2—C13A—C14A	38 (2)	C25—C26—C27—C28	1.2 (13)
C12B—Fe2—C13A—C14A	100 (2)	C26—C27—C28—C23	0.1 (12)
C13B—Fe2—C13A—C14A	78 (4)	C24—C23—C28—C27	−0.7 (11)
C20—Fe2—C13A—C14A	−72 (2)	N3—C23—C28—C27	173.9 (7)
C11A—Fe2—C13A—C14A	85 (2)	N5—N4—C29—C20	0.5 (13)
C19—Fe2—C13A—C14A	−107 (2)	Zn1—N4—C29—C20	−174.7 (7)
C11B—Fe2—C13A—C14A	70 (2)	C19—C20—C29—N4	−13.2 (17)
C17—Fe2—C13A—C14A	−179 (2)	C16—C20—C29—N4	169.6 (9)
C14B—Fe2—C13A—C14A	8(4)	Fe2—C20—C29—N4	−105.6 (10)
C12A—Fe2—C13A—C14A	121 (3)	N4—N5—C30—N6	−178.1 (6)
C12A—C13A—C14A—C15A	0(5)	N4—N5—C30—S2	4.2 (10)
Fe2—C13A—C14A—C15A	−58 (4)	C31—N6—C30—N5	3.3 (12)
C12A—C13A—C14A—Fe2	58 (2)	C31—N6—C30—S2	−178.7 (6)
C12B—Fe2—C14A—C15A	61.4 (19)	Zn1—S2—C30—N5	−12.0 (7)
C13B—Fe2—C14A—C15A	94 (3)	Zn1—S2—C30—N6	170.2 (5)
C20—Fe2—C14A—C15A	−110.9 (17)	C30—N6—C31—C36	−148.8 (8)
C16—Fe2—C14A—C15A	−73 (3)	C30—N6—C31—C32	32.7 (12)
C11A—Fe2—C14A—C15A	41.1 (17)	C36—C31—C32—C33	3.2 (12)
C19—Fe2—C14A—C15A	−154.3 (15)	N6—C31—C32—C33	−178.4 (8)
C11B—Fe2—C14A—C15A	17.9 (17)	C31—C32—C33—C34	−2.8 (12)
C14B—Fe2—C14A—C15A	2(20)	C32—C33—C34—C35	1.4 (14)
C12A—Fe2—C14A—C15A	84.6 (19)	C33—C34—C35—C36	−0.4 (14)
C15A—Fe2—C14A—C13A	−120 (3)	C34—C35—C36—C31	0.8 (14)
C12B—Fe2—C14A—C13A	−58.5 (19)	C32—C31—C36—C35	−2.1 (14)
C13B—Fe2—C14A—C13A	−26.3 (14)	N6—C31—C36—C35	179.3 (7)

### Hydrogen-bond geometry (Å, °)

**Table:**

*D*—H···*A*	*D*—H	H···*A*	*D*···*A*	*D*—H···*A*
O1W—H2W1···S1^i^	0.85	2.61	3.238 (10)	132
N3—H3B···O1W^ii^	0.86	2.14	2.981 (10)	165
N6—H6B···O1W	0.86	2.12	2.927 (11)	155
C18—H18A···N2^iii^	0.98	2.60	3.455 (10)	146
O1W—H1W1···Cg1^iv^	0.85	2.63	3.164 (9)	123

Symmetry codes: (i) *x*, *y*+1, *z*; (ii) *x*, *y*−1, *z*; (iii) −*x*+1, *y*+1/2, −*z*+1; (iv) −*x*+1, *y*+1/2, −*z*.

## References

[bb1] Bruker (2005). *APEX2*, *SAINT* and *SADABS*. Bruker AXS Inc., Madison, Wisconsin, USA.

[bb2] Casas, J. S., Castaño, M. V., Cifuentes, M. C., Garcia-Monteagudo, J. C., Sánchez, A., Sordo, J. & Abram, U. (2004). *J. Inorg. Biochem.* **98**, 1009–1016. 10.1016/j.jinorgbio.2004.02.019 15149809

[bb3] Cosier, J. & Glazer, A. M. (1986). *J. Appl. Cryst.* **19**, 105–107.

[bb4] Flack, H. D. (1983). *Acta Cryst.* A**39**, 876–881.

[bb5] Sheldrick, G. M. (2008). *Acta Cryst.* A**64**, 112–122. 10.1107/S0108767307043930 18156677

[bb6] Spek, A. L. (2009). *Acta Cryst.* D**65**, 148–155. 10.1107/S090744490804362X PMC2631630 19171970

[bb7] Vikneswaran, M. R., Teoh, S. G., Quah, C. K. & Fun, H.-K. (2009*a*). *Acta Cryst.* E**65**, m1027. 10.1107/S1600536809030086 PMC2969868 21577394

[bb8] Vikneswaran, M. R., Teoh, S. G., Razak, I. A. & Fun, H.-K. (2009*b*). *Acta Cryst.* E**65**, m373–m374. 10.1107/S1600536809007363 PMC2969079 21582327

